# Preparation and Tribological Properties of Modified MoS_2_/SiC/Epoxy Composites

**DOI:** 10.3390/ma14071731

**Published:** 2021-04-01

**Authors:** Cheng Liu, Meijuan Li, Qiang Shen, Haikun Chen

**Affiliations:** 1 School of Chemistry, Chemical Engineering and Life Science, Wuhan University of Technology, Wuhan 430070, Hubei, China; LC58010@163.com; 2 State Key Laboratory of Advanced Technology for Material Synthesis and Processing, Wuhan University of Technology, Wuhan 430070, Hubei, China; 3 Science and Technology of Advanced Functional Composites Laboratory, Aerospace Research Institute of Materials & Processing Technology, Beijing 100076, China; haikunc@163.com

**Keywords:** molybdenum disulfide, silicon carbide, surface modification, epoxy matrix composite, tribological properties

## Abstract

In order to improve the tribological properties of epoxy (EP), EP composites were prepared by filling different proportions of silicon carbide (SiC) particles and molybdenum disulfide (MoS_2_) powder. SiC and MoS_2_ particle surfaces were modified by the silane coupling agent KH560 to improve dispersion and avoid agglomeration of the inorganic particles in the EP resin matrix. The effect of different proportions of modified MoS_2_ content on the tribological properties of SiC/EP composites, and the wear mechanism of the worn surface, were investigated when the filler content was fixed at 55 wt.%. The results indicate that the friction and wear properties of modified MoS_2_/SiC/EP composites are better than SiC/EP composites without modified MoS_2_. When the modified MoS_2_ content is 4 wt.%, the average friction coefficient and volume wear rate of the modified MoS_2_/SiC/EP composite are 0.447 and 14.39 × 10^−5^ mm^3^/N·m, respectively, which is reduced by 10.06% and 52.13% in comparison with that of the 55 wt.% SiC/EP composite. Furthermore, the average friction coefficient of a composite containing 4 wt.% MoS_2_ is 16.14% lower, and the volume wear rate is 92.84% lower than that of pure EP.

## 1. Introduction

Epoxy (EP) is one of the most commonly used industrial materials. Owing to its excellent properties, such as high adhesive capacity, excellent chemical stability, and good solvent resistance, it is a very widely used thermosetting resin [1,2]. It has been widely reported that the addition of inorganic powders, such as silicon carbide (SiC), SiO_2_, graphite, etc., can significantly improve the mechanical properties, heat resistance and wear resistance of EP [3,4,5]. However, due to its three-dimensional network structure and poor surface properties, EP cannot be directly used as a wear-resistant material [6]. In order to strengthen and improve the tribological properties of EP, adding different fillers into the EP matrix has become an important and valid approach for solving these problems [7,8]. Most notably, the composite filling of hard particles and solid lubricants is very effective at improving the properties of EP composites, because the complementary properties of these different fillers, and the synergistic effect between fillers, make the tribological properties of EP composites more effective. For example, graphite/glass-EP composites reinforced by SiC particles can obtain better wear resistance [9], and an EP composite with TiO_2_ and graphite fillers together has a lower friction coefficient and better wear resistance than a single filled EP, under all loads and speed conditions [10].

SiC has many excellent properties, such as high hardness, high wear resistance, high mechanical strength, and so on. It can be used to enhance the mechanical properties and tribological properties of polymer composites [11,12,13]. Molybdenum disulfide (MoS_2_) has a special layered structure, similar to graphite, which can enhance the various properties of polymer composites. They can not only improve the mechanical and electrical properties but can also act as a solid lubricant [14,15,16,17]. The layered structure is connected by a weak van der Waals force between the layers, which has good lubricity, making it possible to form a lubricating film on the surface of the polymer and, therefore, improving the friction and wear performance of the polymer composite [18,19].

There is much research on the friction and wear of epoxy resin filled with SiC or MoS_2_ [20,21,22,23,24], but, so far, there are few reports on the synergistic filling of SiC and MoS_2_. In this paper, EP composites with different proportions of SiC and MoS_2_ were prepared. Due to the significant differences in chemical structure and physical form, and a lack of affinity between inorganic fillers and EP resin, surface modification of fillers is essential to make them combine better with polymer and have good dispersion, especially when the filler content is higher, which cannot be well dispersed in the polymer, even through mechanical stirring [25,26]. Therefore, an important silane coupling agent, KH560 (c-glycidoxy propyl trimethoxy silane), is used to form a layer of single molecule membrane on the surface of fillers and improve their wettability and dispersion. There are many reports that focus on the effects of KH560-modified SiC [27,28], but the research on KH560-modified MoS_2_ in relation to the tribological properties of reinforced composites is scarce. In our work, the effect of surface modification by KH560 on MoS_2_ powder was studied in detail. At the same time, KH560-MoS_2_ was added into an EP composite reinforced with a high content of silane coupling agent, KH560-modified SiC powder. The effect of SiC and MoS_2_ on the friction and wear properties of EP composites was studied, and the worn surface morphology of EP composites was analyzed. On this basis, the wear mechanism of SiC and MoS_2_ synergistically reinforced EP composites was discussed, which provided a reference for the high wear resistance design and application of EP composites.

## 2. Materials and Methods

### 2.1. Materials

The experimental reagents mainly included MoS_2_ powder (particle size ≤ 2 μm, 99.8% grad, Shanghai Aladdin Biochemical Technology Co., Ltd., Shanghai, China), SiC powder (D50 = 0.9 μm, 99.5% grad, Shanghai Yaotian New Material Technology Co., Ltd., Shanghai, China), epoxy E51 (Hangzhou wuhuigang adhesive Co., Ltd., Hangzhou, China), curing agent polyethylene polyamine (Aladdin, Shanghai, China), silane coupling agent, KH560, acetone, ethanol, and acetic acid (all purchased from Sinopharm Chemical Reagent Co., Ltd., Shanghai, China).

### 2.2. Preparation of KH560-MoS_2_

Firstly, MoS_2_ and KH560 were mixed in an ethanol and water solvent with proper prescription (pH~4) in a reactor. Then, the mixture reacted for 6 h, with stirring at 60 °C and ultrasonic dispersion for 30 min. After cooling to room temperature, the modified MoS_2_ was centrifugally washed 3 times using ethanol, and, finally, it was dried in a vacuum for 8 h to remove the solvent at 120 °C.

### 2.3. Composites Preparation

**Figure 1:**
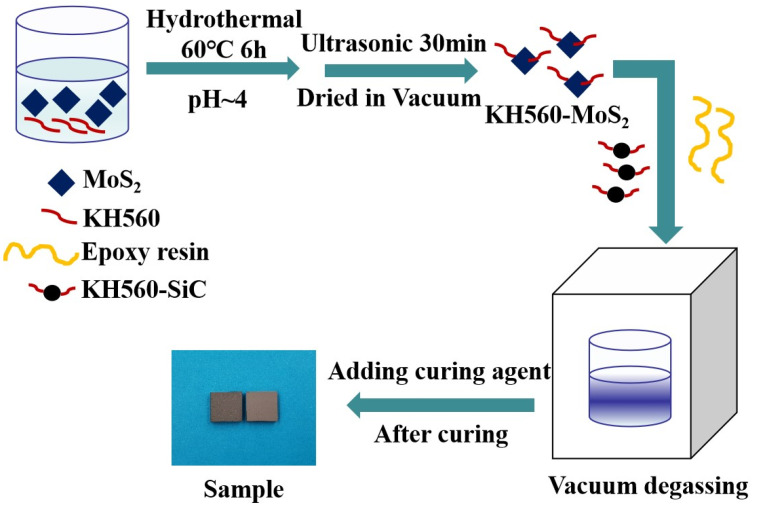
Diagrammatic representation of modified MoS_2_/SiC/EP composites’ preparation.

The as-prepared KH560-MoS_2_ was added into the preheated EP and stirred for 1 h. Then, the KH560-SiC and a little acetone was added into the mixture, stirred, and defoamed at 70 °C under a vacuum for 2 h. After that, the polyethylene polyamine was added into the mixture by slowly stirring. Then, the suspension was poured into the preheated mold and cured in the blast air oven. The selected curing process was 40 °C/12 h + 60 °C/2 h + 100 °C/4 h. Finally, the EP composites were obtained after demoulding. The ingredients of EP composites are listed in Table 1, and the entire synthetic approach of modified MoS_2_/SiC/EP composites is shown in Figure 1.

### 2.4. Measurement and Characterization

A thermal-gravimetric analyzer (TGA) measurement was performed on an STA449F3 instrument (Netzsch instruments, Selb, Germany). About 3.5 mg of sample was put in an alumina crucible and the heating rate was set as 10 °C/min, with the test range from room temperature to 800 °C at argon atmosphere. A Raman laser spectroscopy measurement was performed using a confocal laser, Raman evolution (LabRam HR Evolution, Paris, France), with excitation provided in back-scattering geometry by a 532 nm laser line in an air-ambient environment. An X-ray photoelectron spectroscopy (XPS, Thermo Scientific Escalab 250Xi, Waltham, MA, USA) was used to characterize the surface chemical composition and elemental content of the sample.

A Vickers hardness of samples was obtained by a digital microhardness tester system (Wilson Tukon 1202, Norwood, MA, USA). The maximum indentation load was 0.1 kg, while the holding time at maximum depth was 15 s. In order to reduce the tolerance, the experiments were conducted at five indents for each sample. A field emission scanning electron microscope (FESEM, FEI, Quanta + FEG250, Hillsboro, OR, USA) was used to observe the fracture morphology and dispersion of the samples. The friction and wear tests were tested by an MFT-4000 multifunctional material surface performance instrument (MFT-4000, Lanzhou Huahui Instrument Technology Co., Ltd., Lanzhou, China), with GCr15 steel balls 5 mm in diameter, as the upper specimens at room temperature. The single reciprocating stroke was 10mm. The speed was 180 mm/min and the time lasted for 30 min. The friction coefficient was recorded by the tester, and the volume wear rate K (mm^3^/N·m) was calculated according to the Formula (1):

(1) $$K=\frac{∆V}{F\times L}$$

where Δ*V* is the wear volume (mm^3^), *F* is the test load (N), and *L* is the total reciprocating stroke (m).

A three-dimensional optical profilometer (Nanovea ST400, Irvine, CA, USA) was used to observe the three-dimensional contour map of wear track, and a field emission ultra-high resolution scanning electron microscope (SEM, Zeiss sigma500, Oberkochen, Germany) was used to analyze the worn surface morphology of samples.

## 3. Results and Discussion

### 3.1. Characterization on The Modification of MoS_2_ Powder by KH560

**Figure 2:**
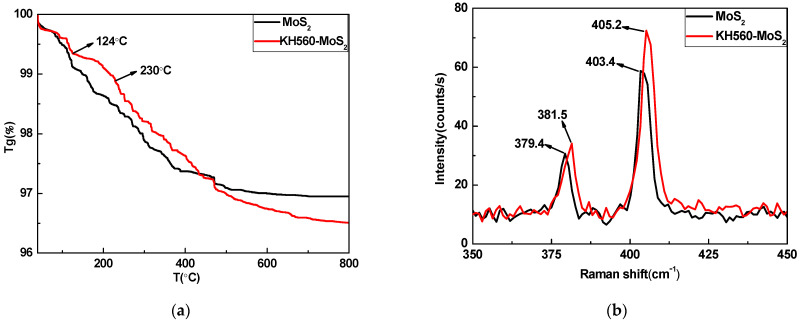
TGA curves (**a**) and Raman Spectroscopy (**b**) of MoS_2_ and KH560-MoS_2_.

The TGA thermal analysis curves of MoS_2_ and KH560-MoS_2_ were shown in Figure 2a. It was seen from the figure that when the temperature started to rise to about 124 °C, the weight loss trend of the two kinds of powders was the same. However, the thermal weight loss of KH560-MoS_2_ was obviously more stable than that of unmodified MoS_2_ in the temperature range of 124 °C~230 °C, which indicated that KH560 successfully modified MoS_2_ and improved the thermal stability of MoS_2_. Overall, the weight loss of modified MoS_2_ was higher than that of unmodified MoS_2_ from room temperature to 800 °C, which was attributed to the decomposition of KH560. A Raman spectroscopy was carried out to garner more structural information, as shown in Figure 2b. Pure MoS_2_ had two dominant peaks, at 379.4 cm^−1^ and 403.4 cm^−1^, which were, respectively, the in-plane $E_{2g}^{1}$ and out-of-plane $A_{g}^{1}$ vibrational modes [29]. It was observed that the $E_{2g}^{1}$ and $A_{g}^{1}$ vibrational modes had different degrees of shift to higher frequencies, and that the shift of $E_{2g}^{1}$ was larger than that of $A_{g}^{1}$, suggesting that the KH560 molecule used for surface modification was combined with MoS_2_ and introduced tensile strain into MoS_2_ layers [30].

**Figure 3:**
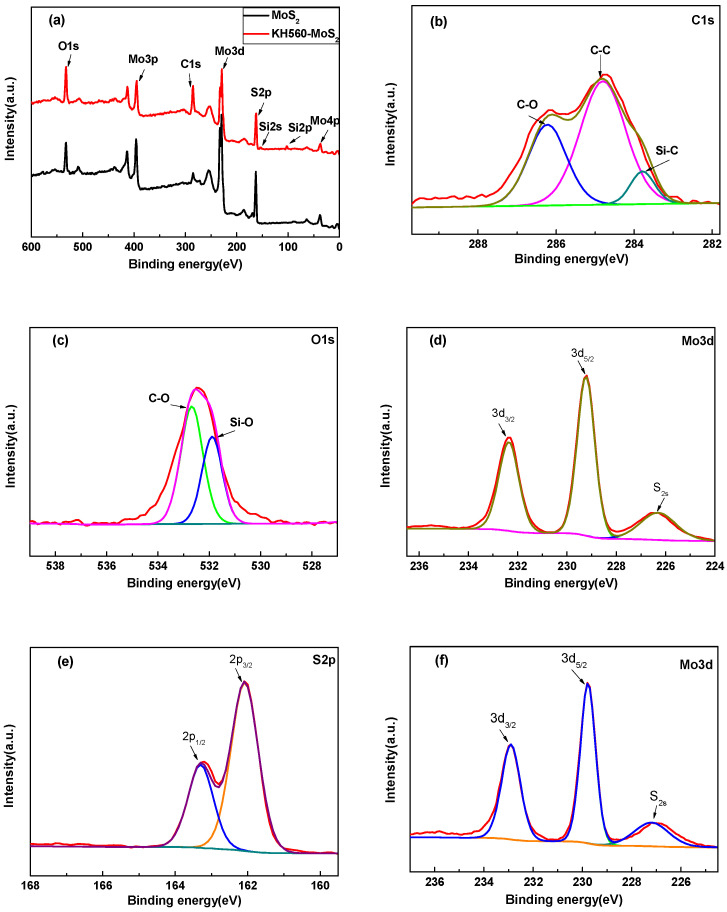

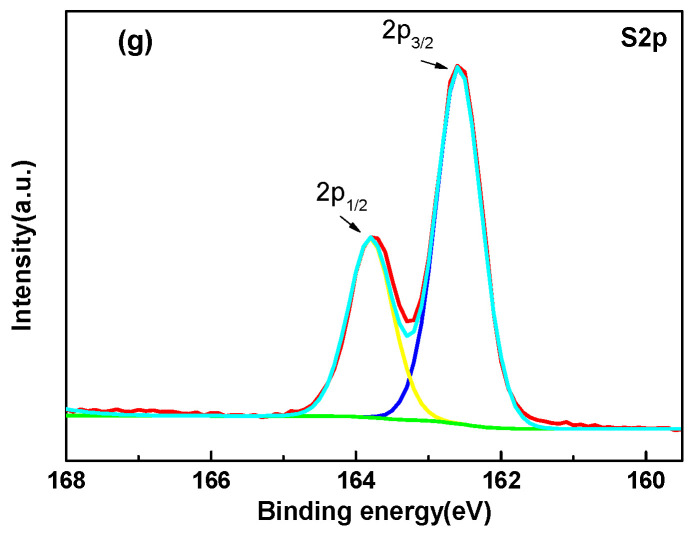
XPS spectra of MoS_2_ and KH560-MoS_2_ (**a**), high-resolution XPS spectra of C1s (**b**); O1s (**c**); Mo3d (**d**); S2p (**e**) for KH560-MoS_2_, and Mo3d (**f**); S2p (**g**) for MoS_2_.

XPS was used to confirm the chemical composition of MoS_2_ and kH560-MoS_2_, as shown in Figure 3. From the full spectrum in Figure 3a, it was found that the peak intensity of C and O elements of the modified MoS_2_ was higher than that of the unmodified MoS_2_, and that the characteristic peaks of silicon (3.76%) appeared in KH560-MoS_2_, indicating that KH560 was grafted onto the MoS_2_ surface successfully. The high-resolution C1s and O1s spectra were to estimate the function groups for KH560-MoS_2_ in Figure 3b,c. The functional groups of C1s peak included C-O, C-C, and Si-C, at 286.22 eV, 284.78 eV, and 283.79 eV, respectively [31,32], and the C-O and Si-O of O1s peaked at 532.68 eV and 531.89 eV, respectively [33,34]. These functional groups were related to KH560 grafted on the surface of MoS_2_. In addition, the high-resolution of Mo3d and S2p for KH560-MoS_2_ and MoS_2_ were studied. Mo3d_3/2_, Mo3d_5/2_, S2p_1/2_, and S2p_3/2_ peaks of KH560-MoS_2_ were located at 232.36 eV, 229.23 eV, 163.30 eV, and 162.08 eV, respectively, and Mo3d_3/2_, Mo3d_5/2_, S2p_1/2_, and S2p_3/2_ peaks of MoS_2_ were located at 232.90 eV, 229.77 eV, 163.80 eV, and 162.58 eV, respectively. It was found that the binding energy of Mo3d and S2p of KH560-MoS_2_ all shifted to lower energy. This could be explained by the electrostatic interaction between MoS_2_ and KH560 [35].

### 3.2. Dispersion of High Content of Modified MoS_2_/SiC in Epoxy Matrix

**Figure 4:**
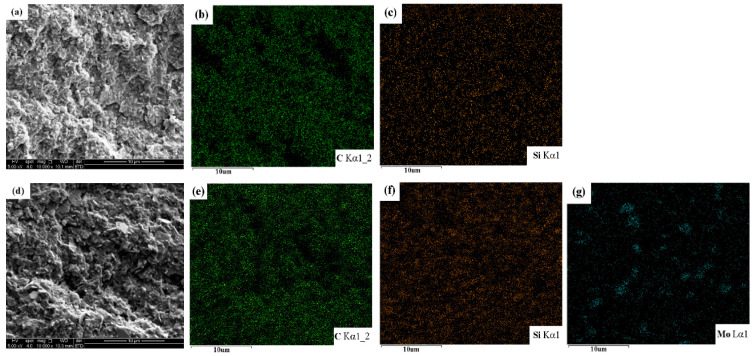
Fracture morphology of S0 (**a**) and EDS results of C (**b**); Si (**c**), and Fracture morphology of S5 (**d**) and EDS results of C (**e**); Si (**f**); Mo (**g**).

The dispersion uniformity of fillers in the epoxy resin matrix directly affects the performance of the EP composites. Most notably, this effect will be more significant when the filler is added in a large proportion [36]. As shown in Figure 4, SEM fracture scanning combined with EDS analysis was used to study the dispersion of high-content-modified SiC/MoS_2_ in EP composites. When the content of KH560-SiC was as high as 55 wt.% (sample S0), it was still evenly distributed in the EP with good dispersibility, and was well combined with the epoxy resin matrix (Figure 4a–c). The total content of inorganic fillers was fixed at 55 wt.%, and KH560-MoS_2_ partially replaced the same mass fraction of KH560-SiC particles; the highest proportion of KH560-MoS_2_ was 5 wt.% (sample S5). SEM and EDS analysis (Figure 4d–g) showed that the modified MoS_2_ with lamellar structure was closely embedded in the modified SiC/EP composites, with uniform distribution and good dispersion. The KH560-MoS_2_ powder size was less than or equal to 2 μm, without agglomeration phenomenon in EP composites, which proved that the MoS_2_ lamellar powder modified by KH560 could also be well dispersed and combined in the epoxy resin matrix.

### 3.3. Tribological Properties of Composites

**Figure 5:**
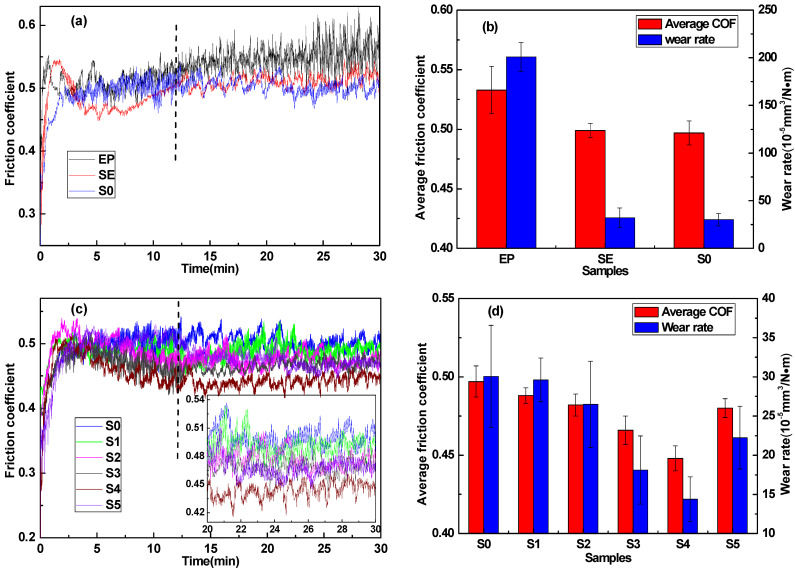
Development of friction coefficient over testing time for EP composites (**a**,**c**), and average COF and wear rate for EP composites at 60 N (**b**,**d**).

**Figure 6:**
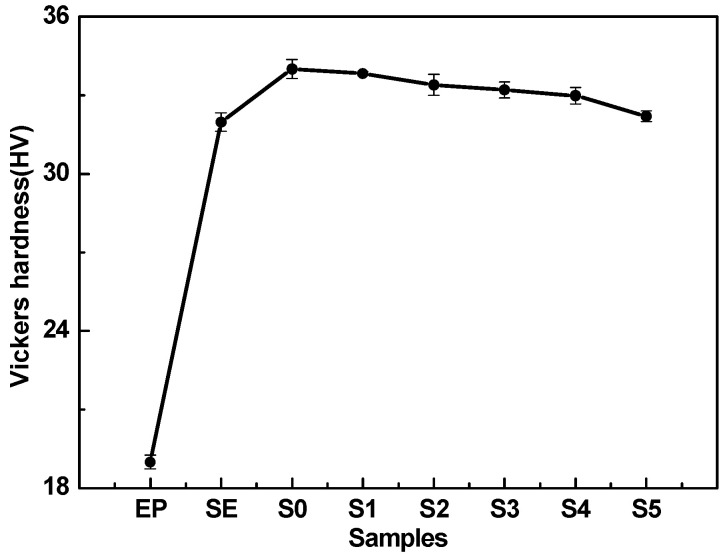
Vickers hardness of EP composites.

The real-time friction coefficient (COF) for the EP and other EP composites, and the resulting variation in the average COF and volume wear rate of composites at 60 N, are shown in Figure 5. It was obvious that all samples had experienced two stages of running-in period and stable period from the friction test curve (Figure 5a,c). In the early stage of the running-in period, the friction coefficients of all samples increased rapidly with the progress of friction. Compared with pure EP, the friction coefficient of EP composites with SiC and MoS_2_ added had relatively small fluctuations in the stable period. In addition, the friction coefficient of pure EP still fluctuated greatly after the stable period. The reason was that, compared with the EP composites, the hardness of pure EP was the lowest, which was 19.0 HV (Figure 6, Vickers hardness of EP and EP composites). The plastic deformation of the EP worn surface was more serious during the friction process, which resulted in serious fatigue wear and which led to a large fluctuation of friction coefficient after the stable period [37]. It was found that when the content of modified SiC increased to 50 and 55 wt.%, the friction coefficient of SE and S0 was lower and tended to be stable [38,39], as shown in Figure 5a. This indicated that the modified SiC particles could be well combined with the epoxy resin matrix, and the matrix stress could be better transmitted to endure higher normal loads when the content of SiC particles was higher. At the same time, a large amount of SiC particles distributed on the friction interface could reduce the tearing of the EP composite surface and the formation of furrows, thereby reducing the friction coefficient [40].

Figure 5b depicts that the average COF of the EP composite adding 55 wt.% SiC particles was 0.497, and the volume wear rate was 30.06 × 10^−5^ mm^3^/N·m. Compared with pure EP, the average COF was reduced by 6.75% from 0.533, and the volume wear rate was reduced by 85.04% from 200.98 × 10^−5^ mm^3^/N·m. If the addition of SiC particles was reduced to 50 wt.% (sample SE), the average COF and volume wear rate had no obvious change compared to sample S0. Therefore, the fixed total content of fillers added in EP was 55 wt.% in the composite material with modified MoS_2_ powder. As shown in the inset of Figure 5c, the friction coefficient of the EP composites with modified MoS_2_ powder in the stable period was lower than that of S0, which proved that the addition of MoS_2_ improved the friction-reducing performance of EP composites better [41]. When the content of modified MoS_2_ was increased to 4 wt.% (Figure 5d, sample S4), the measured lowest average COF was 0.447, which was reduced by 10.06% compared to 0.497 of S0, and was reduced by 16.14% compared to 0.533 of EP. At the same time, the measured lowest volume wear rate was 14.39 × 10^−5^ mm^3^/N·m of S4, which was reduced by 52.13% compared to 30.06 × 10^−5^ mm^3^/N·m of S0, and was reduced by 92.84% compared to 200.98 × 10^−5^ mm^3^/N·m of EP. When the content of modified MoS_2_ continued to increase, the average COF and volume wear rate of the modified MoS_2_/SiC/EP composites increased; but they were still lower than those of S0. With reference to Figure 6, as the content of modified MoS_2_ increased, the hardness of the EP composites decreased. When the content of modified MoS_2_ was 5 wt.% (S5), the hardness of modified MoS_2_/SiC/EP composite was the lowest, which was 32.2HV. Although the modified SiC and MoS_2_ could be well dispersed in the resin matrix, the composite hardness would be reduced when MoS_2_ replaced the same amount of SiC. This is because the hardness of SiC particles is higher than that of MoS_2_, and some MoS_2_ particles had larger particle size, smaller specific surface area, and lower surface energy, which made the bonding strength between MoS_2_ and epoxy resin weak. In this case, the contact area of the friction pair on the surface of the composites increased, and more hard abrasive SiC particles were produced in the early stage of the running-in period, which resulted in slight abrasive wear during the friction process and led to the increase of the friction coefficient and the volume wear rate.

**Figure 7:**
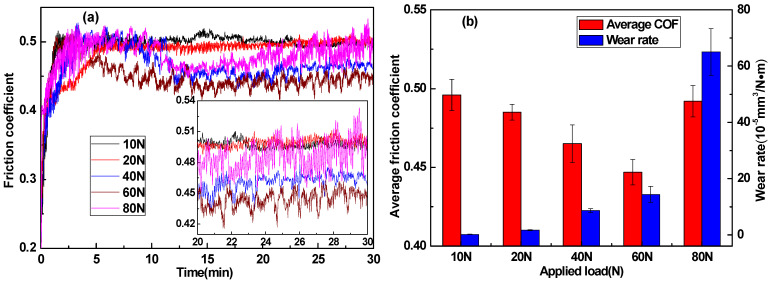
Development of friction coefficient over testing time for S4 (**a**), and average COF and wear rate for S4 at different applied loads (**b**).

Figure 7 showed the effect of applied load on the friction coefficient and wear rate of modified MoS_2_/SiC reinforced EP composites, and the test sample was S4. As shown in Figure 7a, when the applied load was only 10~20 N, the friction curve of the stable period tends to be flat and straight, indicating that the friction process was proceeding on its surface. The wear rate was very small, and a small number of particles adhered to the dual surface. When the applied load was gradually increased to 40~60 N, the MoS_2_ particles with weak bonding interface, bonded by van der Waals force between the layers, would extrude and penetrate into the matrix, resulting in the formation of lubricating film at the friction interface, which reduced the friction resistance and decreased the friction coefficient gradually. However, with the applied load increasing to 80 N, the lubricating film produced by MoS_2_ particles could reduce the friction coefficient in the middle stage of friction. The friction coefficient began to fluctuate greatly in the late stage after 20 min (shown in the inset of Figure 7a), resulting in the damage of the lubricating film on the friction interface and severe fatigue wear, which led to the obvious increase of the friction coefficient and volume wear rate (Figure 7b) [42].

### 3.4. Wear Mechanism Analysis of Composites

**Figure 8:**
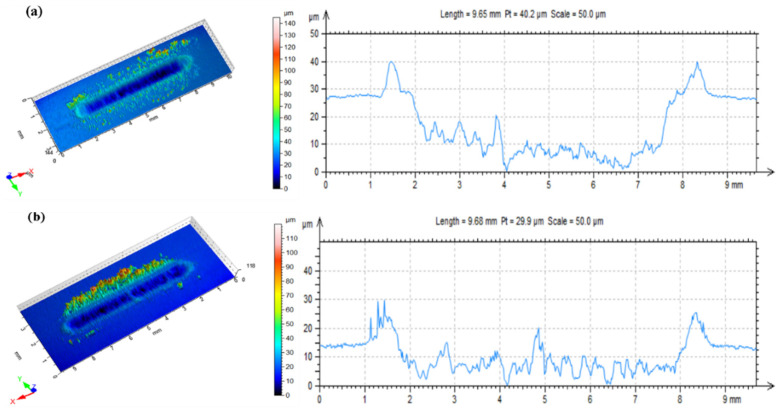
Three-dimensional contour map of wear track of S0 (**a**) and S5 (**b**) at 60 N.

Comparing the three-dimensional profile of S0 and S4 samples after the friction test at 60 N (shown in Figure 8), and combining it with the hardness test results (shown in Figure 6), it can be seen that when the additional amount of modified MoS_2_ is 4 wt.%, the depth of the wear scar is relatively shallow and the depth distribution of the wear scar is relatively consistent; although, the hardness of EP composites is lower than that of 55 wt.% SiC/EP composites without modified MoS_2_. This illustrates that the soft phase addition of MoS_2_ can reduce the hardness of the composites, and the addition of properly modified MoS_2_ can not only improve the composite’s ability to withstand applied loads, but, also, spread on the friction surface, thereby effectively reducing the actual friction area and playing a better role in wear resistance [43].

**Figure 9:**
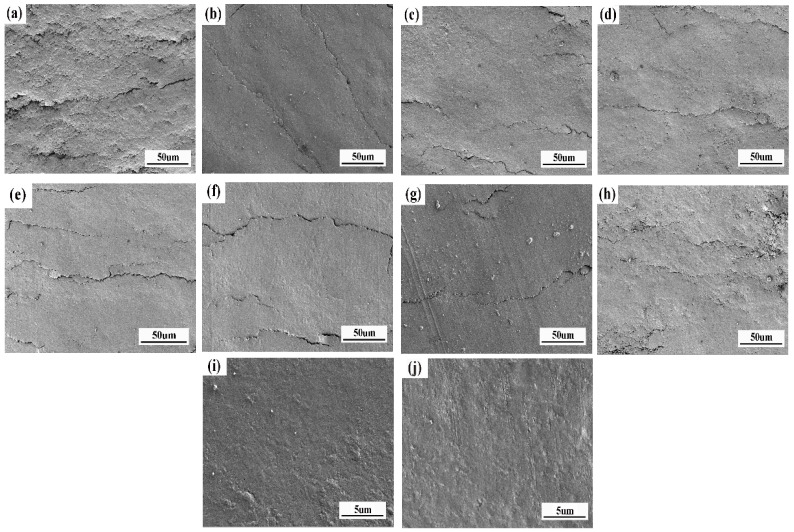
SEM images of wear track on the composites EP (**a**); S0 (**b**,**i**); S1 (**c**); S2 (**d**); S3 (**e**); S4 (**f**,**h j**); S5 (**g**). The load of S4 (**h**) is 80 N, while the others are 60 N.

In order to investigate the wear mechanism for filled and unfilled composites, the corresponding low and high magnification SEM images of the worn surfaces are demonstrated in Figure 9. The worn surface of EP (Figure 9a) appears rough and more microcracked. This indicates that part of the EP is detached and the friction damage to the material is more serious, showing typical fatigue wear characteristics. For the composite with 55 wt.% modified SiC, the worn surface is relatively smooth, and some hard abrasive SiC particles are produced (Figure 9b). In addition, the hard abrasive SiC particles can be seen protruding on the worn surface at higher SEM magnification (Figure 9i), which shows the abrasive wear mechanism. After adding appropriately modified MoS_2_, which lends solid lubricant properties, the MoS_2_ particles evenly dispersed in the composites gradually migrate to the worn surface during the friction process, and provide a lubrication effect, thus, making the worn surface smoother (Figure 9c–f). In addition, it seems that the transfer film has a compact and smooth structure; this can be seen clearly in a high SEM magnification (Figure 9j). When the content of modified MoS_2_ increases to 5 wt.% (Figure 9g), some hard abrasive SiC particles exist on the worn surface, resulting in slight abrasive wear, which increases the friction coefficient and volume wear rate. Nevertheless, with the normal load increases exceeding the capacity of the modified MoS_2_/SiC/EP composite, the lubricant film will be partially destroyed, and more microcracks appear on the worn surface, as shown in Figure 9h. At this moment, the main wear mechanism is fatigue wear, which greatly increases volume wear rate.

## 4. Conclusions

The surface of MoS_2_ particles was successfully modified by silane coupling agent KH560 to improve its dispersion in EP composites. An MFT-4000 multifunctional material surface performance tester was used to evaluate the friction and wear properties of the composites filled with modified MoS_2,_ instead of SiC, with the same mass fraction when the fixed filler content was 55 wt.%. The wear mechanism was discussed by analyzing the worn surface. Our main conclusions could be drawn as follows:

(1) The results of TGA, Raman and XPS showed that the surface of MoS_2_ particles was successfully modified by KH560.
(2) It is found that the friction and wear properties of SiC/EP composites with MoS_2_ are better than those without MoS_2_. When the adding fraction of modified MoS_2_ is 4 wt.%, the friction coefficient and volume wear rate of EP composites are lowest. Compared with 55 wt.% SiC/EP composite, the friction coefficient and volume wear rate of the SiC/EP composite containing 4 wt.% MoS_2_ are reduced by 10.06% and 52.13%, respectively. Furthermore, the composite friction coefficient is decreased by about 16.14%, from 0.533 to 0.447, and the volume wear rate is reduced by 92.84%, from 200.98 × 10^−5^ mm^3^/N·m to 14.39 × 10^−5^ mm^3^/N·m, in comparison with those of pure EP, proving the significant improvement of wear resistance of EP composites.
(3) When the appropriate content of MoS_2_ with lubricating properties is added, the MoS_2_ homogenously dispersed in the material gradually migrates to the surface during the friction process to form the lubricating film, so as to make the worn surface smoother. However, when the weak-bonded MoS_2_ content increased to 5 wt.%, some hard abrasive SiC particles existed on the wear surface, resulting in slight abrasive wear, which increased the friction coefficient and volume wear rate. In addition, if the load increases and exceeds the capacity of the modified MoS_2_/SiC/EP composite, the lubricant film is partially destroyed, and the main wear mechanism is fatigue wear.

## Figures and Tables

**Table 1:** Ingredients of epoxy composites.

Sample	Fillers (wt.%)		EP (wt.%)
	SiC	MoS_2_	
EP	0	0	100
SE	50	0	50
S0	55	0	45
S1	54	1	45
S2	53	2	45
S3	52	3	45
S4	51	4	45
S5	50	5	45

## Data Availability

Data sharing not applicable.

## References

[1] Ahmadi-Khaneghah A., Omidi-Ghallemohamadi M., Behniafar H. (2019). PEG-based epoxy and epoxy/silica networks: Thermal, mechanical, and thermo-mechanical investigations. Int. J. Adhes. Adhes..

[2] Guo Q.B., Lau K.T., Rong M.Z., Zhang M.Q. (2010). Optimization of tribological and mechanical properties of epoxy through hybrid filling. Wear.

[3] Hwang Y., Kim M., Kim J. (2014). Fabrication of surface-treated SiC/epoxy composites through a wetting method for enhanced thermal and mechanical properties. Chem. Eng. J..

[4] Abenojar J., Tutor J., Ballesteros Y., Real J.C., Martinez M.A. (2017). Erosion-wear, mechanical and thermal properties of silica filled epoxy nanocomposites. Compos. Part B.

[5] Yasmin A., Daniel I.M. (2004). Mechanical and thermal properties of graphite platelet/epoxy composites. Polymer.

[6] Dasari A., Yu Z.Z., Mai Y.W. (2009). Fundamental aspects and recent progress on wear/scratch damage in polymer nanocomposites. Mater. Sci. Eng. R..

[7] Shen X.J., Pei X.Q., Liu Y., Fu S.Y. (2014). Tribological performance of carbon nanotube-graphene oxide hybrid/epoxy composites. Compos. Part B.

[8] Baptista R., Mendão A., Rodrigues F., Figueiredo-Pina C.G., Guedes M., Marat-Mendes R. (2016). Effect of high graphite filler contents on the mechanical and tribological failure behavior of epoxy matrix composites. Theor. Appl. Fract. Mech..

[9] Basavarajappa S., Ellangovan S. (2012). Dry sliding wear characteristics of glass-epoxy composite filled with silicon carbide and graphite particles. Wear.

[10] Xian G.J., Walter R., Haupert F. (2006). A synergistic effect of nano-TiO_2_ and graphite on the tribological performance of epoxy matrix composites. J. Appl. Polym. Sci..

[11] Nhuapeng W., Thamjaree W., Kumfu S., Singjai P., Tunkasiri T. (2008). Fabrication and mechanical properties of silicon carbide nanowires/epoxy resin composites. Curr. Appl. Phys..

[12] Chisholm N., Mahfuz H., Rangari V.K., Ashfaq A., Jeelani S. (2005). Fabrication and mechanical characterization of carbon/SiC-epoxy nanocomposites. Compos. Struct..

[13] Zhou T.L., Wang X., Gu M.Y., Xiong D.S. (2009). Study on Mechanical, Thermal and Electrical Characterizations of Nano-SiC/Epoxy Composites. Polym. J..

[14] Kumar V., Monika, Lee D.J. (2020). High-actuation displacement with high flexibility for silicone rubber and few layer graphene composites. Sens. Actuators A..

[15] Kumar V., Lee G., Singh K., Choi J., Lee D.J. (2020). Structure-property relationship in silicone rubber nanocomposites reinforced with carbon nanomaterials for sensors and actuators. Sens. Actuators A..

[16] Kumar V., Alam M.N., Manikkavel A., Choi J., Lee D.J. (2020). Investigation of silicone rubber composites reinforced with carbon nanotube, nanographite, their hybrid and applications for flexible devices. J. Vinyl. Addit. Technol..

[17] Chen B., Ni B.J., Liu W.T., Ye Q.Y., Liu S.Y., Zhang H.X., Yoon K.B. (2018). Mechanical properties of epoxy nanocomposites filled with melamine functionalized molybdenum disulfide. RSC Adv..

[18] Pettarin V., Churruca M.J., Felhs D., Karger-kocsis J., Frontini P.M. (2010). Changes in tribological performance of high molecular weight high density polyethylene induced by the addition of molybdenum disulphide particles. Wear.

[19] Alajmi M., Alrashdan K.R., Alsaeed T., Shalwan A. (2020). Tribological characteristics of graphite epoxy composites using adhesive wear experiments. J. Mater. Res. Technol..

[20] Raj V.R., Ramnath B.V. (2020). Mechanical, Thermal and Wear Behavior of SiC Particle Strengthening of PMMA-Toughened Glass-Epoxy Hybrid Composite. Silicon.

[21] Dass K., Chauhan S.R., Gaur B. (2015). Evaluation of Mechanical, Friction, and Wear Characteristics of Nano-SiC Filled Ortho Cresol Novalac Epoxy Composites under Dry Sliding Condition. Adv. Polym. Technol..

[22] Bhagyashekar M.S., Rao R.M.V.G.K. (2007). Effects of Material and Test Parameters on the Wear Behavior of Particulate Filled Composites Part 1: SiC-Epoxy and Gr-Epoxy Composites. J. Reinf. Plast. Compos..

[23] Sudheer M., Karthik M.N., Kewin A.M., Jonthan B., Mayur J.K. (2013). Mechanical and Abrasive Wear Behavior of Metal Sulphide Lubricant Filled Epoxy Composites. ISRN Polym. Sci..

[24] Srivastava S.K., Sahoo A.K., Bindumadhavan K., Manu S.K., Nayak B.B., Biswas K., Saxena A.K., Singh R. (2010). Reinforcement of ball shaped MoS_2_ nanoparticles in epoxy resin. J. Nanosci. Nanotechnol..

[25] Li Y.C., Wang Z.Y., Zhan Y.H., Wang S.S., Tao X.Q., Liao C.Z., Lu Z.G. (2019). Improved Mechanical and Dielectric Performances of Epoxy Nanocomposites Filled with Aminated Polyethylene Glycol Grafted Graphene. Mater. Lett..

[26] Shang X.J., Zhu Y.M., Li Z.H. (2017). Surface modification of silicon carbide with silane coupling agent and hexadecyl iodiele. Appl. Surf. Sci..

[27] Gu J.W., Zhang Q.Y., Dang J., Zhang J.P., Chen S.J. (2009). Preparation and mechanical properties researches of silane coupling reagent modified β-silicon carbide filled epoxy composites. Polym. Bull..

[28] He Y., Chen Z.C., Ma L.X. (2010). Thermal Conductivity and Mechanical Properties of Silicone Rubber Filled with Different Particle Sized SiC. Adv. Mater. Res..

[29] Li H., Zhang Q., Yap C.C.R., Tay B.K., Edwin T.H.T., Olivier A., Baillargeat D. (2012). From Bulk to Monolayer MoS_2_: Evolution of Raman Scattering. Adv. Funct. Mater..

[30] Wang D., Wen P.Y., Wang J., Song L., Hu Y. (2017). The effect of defect-rich molybdenum disulfide nanosheets with phosphorus, nitrogen and silicon elements on mechanical, thermal, and fire behaviors of unsaturated polyester composites. Chem. Eng. J..

[31] Wang M.Y., Ma L.C., Shi L.L., Feng P.F., Wang X.J., Zhu Y.Y., Wu G.S., Song G.J. (2019). Chemical grafting of nano-SiO_2_ onto graphene oxide via thiol-ene click chemistry and its effect on the interfacial and mechanical properties of GO/epoxy composites. Compos. Sci. Technol..

[32] Jing Y.J., Wang P.Q., Yang Q.B., He Y., Bai Y. (2019). The effect of a functionalized defect-rich molybdenum disulfide nanosheets on anticorrosion performance of epoxy coating. Mater. Res. Express..

[33] Rangarajan S., Aswath P.B. (2011). Role of precursor chemistry on synthesis of Si-O-C and Si-O-C-N ceramics by polymer pyrolysis. J. Mater. Sci..

[34] Kelemen S.R., Afeworki M., Gorbaty M.L., Cohen A.D. (2002). Characterization of Organically Bound Oxygen Forms in Lignites, Peats, and Pyrolyzed Peats by X-ray Photoelectron Spectroscopy (XPS) and Solid-State 13C NMR Methods. Energy Fuels..

[35] Zhou K.Q., Gao R., Qian X.D. (2017). Self-assembly of exfoliated molybdenum disulfide (MoS_2_) nanosheets and layered double hydroxide (LDH): Towards reducing fire hazards of epoxy. J. Hazard. Mater..

[36] Jiao W.Z., Liu Y.Z., Qi G.S. (2009). Studies on mechanical properties of epoxy composites filled with the grafted particles PGMA/Al_2_O_3_. Compos. Sci. Technol..

[37] Belon C., Schmitt M., Bistac S., Croutxé-Barghorn C., Chemtob A. (2011). Friction and wear properties of hybrid sol-gel nanocomposite coatings against steel: Influence of their intrinsic properties. Appl. Surf. Sci..

[38] Luo Y., Rong M.Z., Zhang M.Q. (2007). Tribological behavior of epoxy composites containing reactive SiC nanoparticles. J. Appl. Polym. Sci..

[39] Durand J.M., Vardavoulias M., Jeandin M. (1995). Role of reinforcing ceramic particles in the wear behaviour of polymer-based model composites. Wear.

[40] Jiang Y.M., Liu K., Tang X.K., Li Z.S. (2018). Preparation and properties of epoxy composite reinforced with SiC. J. Synth. Cryst..

[41] Zalaznik M., Novak S., Huski M., Kalin M. (2016). Tribological behaviour of a PEEK polymer containing solid MoS2 lubricants. Lubri. Sci..

[42] Hao Y., Zhou X.Y., Shao J.J., Zhu Y.K. (2019). The influence of multiple fillers on friction and wear behavior of epoxy composite coatings. Surf. Coat. Technol..

[43] Liu Z., Jia Y., Zhao C.B., Han M., Yang J.X. (2019). Preparation of modified MoS_2_/bismaleimide resin and research on its properties. China Molybdenum Ind..

